# Foreign

**Published:** 1840-10-10

**Authors:** 


### FOREIGN.

*On the Operation for Strabismus.* By Professor Dieffenbach.—Since my first communication on this operation, it has had such a general reception, and has acquired such an importance as I did not at that time anticipate. Upwards of three hundred cases have been operated on by me within a few months, and both in Berlin and in other places my proceeding has been frequently imitated. I propose to give here a short general view of the results of my observations.

The youngest individuals in whom I have undertaken the division of the shortened muscle of the eye were five years old; the oldest were upwards of forty.

Sometimes one, sometimes both eyes squinted, and the operation had generally the same favourable result in both cases. When both eyes were affected, I either operated first on that which squinted most, and when that was quite well on the other, or else on both at the same time.

Squinting inwards, from shortening of the rectus internus, was by far most frequent. Sometimes the trochlearis muscle was also shortened, so that it was necessary to divide it as well as the rectus. In the whole number of those I operated on there were only a few who squinted outwards, and still fewer in whom the eye was directed upwards, or upwards and inwards. I found no eyes at all that squinted downwards.

Strabismus upwards was sometimes complicated with blepharoptosis. The division of the rectus superior not only cured the squinting, but the ptosis gradually diminished after it.

Strabismus outwards or inwards was often complicated with nystagmus bulbi. After the division of the external or internal rectus, not only did the squinting cease, but in general the nystagmus also. In other cases, however, the latter was persistent, and did not decrease till after the division of the rectus superior, or obliquus superior, or rectus externus.

When cataract and strabismus co-existed, the operations for both were done at the same time, and the result was in every case favourable to both.

In most of the patients the strabismus had commenced in very early childhood after ophthalmia neonatorum, scrofulous inflammation of the eyes with ulcers on the cornea, or after acute exanthemata, &c. In many there were cicatrices on the cornea or cataracta centralis. In cases of the former kind, in which hitherto artificial pupils would have been made, the operation was attended by success and considerable improvement of the sight.

All those who had strabismus of only one eye saw more weakly with it than with the other; in those who squinted with both eyes, that which was turned least, was usually the stronger. The weakness of the one eye had been observed by only a few of the patients; they had naturally looked only with the better eye, and the other had been unemployed. The operation completely cured the weakness of sight; some who had actually amaurotic amblyopia could see clearly directly after it was performed.

Some of the patients, previous to the operation, often saw double; this defect continued for some time after it, and then gradually ceased. Some others who had never seen double before did so immediately after the operation. These had been in the habit of looking only with their strong eye while the other had been unused. The improved position of the latter compelled it to see; but the double vision was subsequently lost.

Some who were operated on did not see so well immediately after as before the operation; but after some exercise this weakness of vision ceased, and they could then see quite clearly. The cause of this was that when the eye was put in its normal position, a point of the retina, which was before unexercised, was now brought into play, and required some practice before it could fully discharge its functions.

*Operation.*—That for strabismus convergens is here taken as the type. The operator always stands on the right side of the patient, whether he be operating on the right or on the left eye. The patient sits on a stool, and an assistant standing behind him draws up the upper eyelid with a Pellier's hook. A second assistant draws down the lower eyelid with a double hook which is set in a handle, and of which the teeth are connected by a transverse piece. He kneels down before the patient so as not to be in the way.

The operator then puts a fine hook into the conjunctiva, at the inner angle of the eye, just where it is passing from the palpebræ to the bulb, passes it superficially through it, and gives it to a third assistant who stands on the left side of the patient. The operator next passes a second hook in the same way through the conjunctiva about a line and a half from the first. He and his assistant then both at the same time draw their hooks a little up, so as to raise a fold of the conjunctiva, and at the same time pull the bulb somewhat outwards. The fold is then divided with a pair of curved eye-scissors; and this cut usually at once exposes the tendon and the anterior part of the muscle. A couple of cuts with the scissors then expose the outer surface of the muscle; a rather blunt hook is passed under its tendon, and the two sharp hooks that held the conjunctiva are now removed; the eye is held completely in the power of the blunt hook, and is to be drawn by it from out the internal angle of the orbit. A flat probe is then pushed under the muscle; and the loose connexion by cellular tissue between it and the eye is broken up. The division of the muscle is made by the scissors already mentioned, either, first, through the tendon in front of the hook; or, second, behind the hook at the beginning of the muscular substance; or, third, some lines deeper back.

When the tendon is divided nothing of it remains on the eye, and the muscle commonly retracts a line backwards. When the muscle itself is divided at its anterior part or further back, its posterior portion retracts, and the anterior, which remains connected with the bulb, turns forwards like a loose flap, which, according to circumstances, may be removed by the scissors, or pushed back into the wound if it is thought desirable that it should unite again with the posterior portion.

In practised hands the whole operation seldom lasts more than a minute; and it is done almost without pain. When finished, the eye is cleaned with cold water and a soft sponge. The after-treatment consists of cold lotions, and very great abstinence from food and strong drinks. The patient should be kept in a darkened room. In most cases the wound heals very quickly; and after a few weeks no traces of the operation remain, and the eye stands in its normal position.

The operation for internal strabismus is by far the most easy; the division of the obliquus superior for squinting upwards and inwards is more difficult; that of the rectus externus for strabismus divergens is more difficult still; and the most difficult of all is the division of the rectus superior for squinting upwards. With respect to the manipulations of these operations, they are just the same as those for strabismus convergens.

*Remarks on the operation.*—The fixing of the upper and lower eyelids with the elevator and the hook, so as to expose the whole of the anterior surface of the globe, is indispensable; for neither the will of the patient, nor the separation of the lids by the finger, can do this effectually.

The fixing of the globe can be accomplished only by fine hooks carried superficially through the conjunctiva; the seizing and elevation of the fold of conjunctiva by forceps, sounds more gentle than to do it with a sharp hook; but it is in reality far more painful, more injurious, and more insecure; the fold raised up by the forceps easily tears or slips from their grasp, and if the forceps are made with hooks, they wound as well as pinch the membrane. Two hooks must be employed to make the fold tense enough.

The great number of operations that I have performed, has given me opportunity of observing the phenomena that ensue subsequently to them, and their after consequences. The question here is only of internal strabismus, but any surgeon will easily supply the necessary modifications for the operations in the other varieties. In the first case, the eye, after the division of the muscle, goes into its normal position. In the second, it remains in some degree squinting. In the third, it turns outwards.

In my first operations, the position of the eye after the division of the muscle, was left to chance; but I gradually succeeded in getting it into my own power to determine it. If there be the slightest degree of convergent strabismus, only a very small opening should be made in the conjunctiva, and the tendon only divided close to the eye without separating the muscle from the globe. In this case the eye at first almost maintains its previous position, but after some weeks it becomes straight. If the conjunctiva be more extensively divided, and if the under surface of the muscle be separated from the globe with a probe and then cut across, the squinting is at once nearly or completely removed. If the conjunctiva be divided over a greater arc and towards the back of the globe, if the cellular tissue be extensively separated, and the muscle be detached far back and divided at its middle, then the eye, even in cases in which the whole cornea was before hidden in the internal angle, stands quite straight after the operation.

Hundreds of those whom I have cured have been seen by our own and by foreign medical men, and it was often impossible to distinguish the eye that had squinted from the other.

In some who still squinted slightly after the operation, the position of the eye was made perfectly normal by tying up the sound eye, and rolling the globe forcibly outwards, so as to stretch the newly-formed uniting substance. In others of those whom I first operated on, in whom I had not adopted this after measure, I repeated the operation; and I found that in fourteen days after its first division the muscle was again intimately united with the globe, and that with the exception of a slight thickening and induration of this part there was no indication of a previous operation.

Immediately after the operation the eye can be moved inwards by the superior oblique, and at a later period by the divided muscle which has again united with the globe. In several persons in whom the muscle was divided deeper in the orbit the eye turned outwards some weeks after the operation, so that there was actually an external strabismus. If the divergence was but slight, it was often sufficient to snip (*betupfen*) the conjunctiva at the inner angle, so as by the shortening of the membrane that was thus produced, to bring the eye into the middle. But if the divergence was greater, I then divided the external rectus, and the eye became straight again, especially when I at the same time removed a fold of conjunctiva from the internal angle, for the cicatrix that then followed tended to draw the globe inwards. If the eye, notwithstanding the division of the external rectus, still remained turned outwards, I then, after dividing and separating that muscle, tied a thread as fine as a hair upon its tendon, and with this pulled the eye forcibly inwards. The end of the thread was then drawn tightly across the bridge of the nose, and fastened to a piece of good adhesive plaster, which was stuck upon the opposite side. The result for the most part surpassed my expectations.—*Brit. and For. Med. Rev.*, *from Casper's Wochenschrift.* July 4, 1840.

---

*On the Operation for Strabismus. Claims of M. Jules Guerin.* By M. Jules Guerin.—In a letter on the treatment of strabismus, addressed to the Academy of Sciences, 29th June, 1840, M. Guerin says: "I have the honour to inform the Academy that I have four times practised with success the division of the muscles of the eye, in cases of convergent strabismus. I shall briefly state the principles which have directed me in this operation. I had long and publicly professed that strabismus was the result of the contraction of the muscles of the eye; and the varieties of this deformity the necessary consequence of different degrees of contraction. It is in fact an application of my general theory of articular deformities in the skeleton, and caused one of the most eminent members of the academy to designate strabismus as *club-foot of the eye.*"

M. Guerin goes on to state that in his clinical lectures he frequently proposed to extend division of the muscles to deviations of the eye in the same manner that he had applied it to deformities of similar origin, and that eighteen months since he offered to cure Dr. Pinel Grandchamp of strabismus by means of this operation. He proceeds: "As for the operation itself, it differs in some respects from that of M. Dieffenbach. One of the reasons which induced me to pause before attempting his method, was the fear of inflammation occurring in a wound exposed to the air upon a delicate organ, and so near the cerebrum. These accidents I feel certain of avoiding by the following method:

"In place of dividing layer after layer, I detach the portion of ocular conjunctiva, which covers the muscles from the sclerotic, and raise it with broad-nibbed forceps until the muscle is exposed. This being divided with curved scissors, I restore the detached portion of conjunctiva to its place; this by closing the wound prevents the access of air, and obtains all the advantages of a subcutaneous wound. Experience has confirmed my preconceived notions of the theory; in the four operations I have performed there has been no sign of suppurative inflammation.

"The result of these operations has been very satisfactory; but not so immediately advantageous as M. Dieffenbach observed. In one instance there was a complete and instantaneous restoration of the natural position of the eye, in the others only an amelioration. This circumstance appears to be a natural consequence of the true origin of strabismus. At one time the deviation of sight is originally muscular, and the result of spasmodic contraction in a single muscle; at another time the contraction is consecutive or even primitive; but it has simultaneously affected many muscles."—*Ibid.*, *from L'Expérience.* Juillet 9, 1840.

---

*On the Cure of Strabismus.* By Dr. F. A. von Ammon.—[In the great number of the papers written on this subject, it is scarcely to be expected that more than two or three remarks on each should be worth abstracting. In the present article by the editor of the Monatsschrift, the following passages are worthy the attention of our readers:]

A principal point is the determination of the indications for the operation; and I think it the more important, because the whole history of the affection, notwithstanding the works of many excellent physiologists, is extremely obscure and uncertain. Nor could this be otherwise when the anormal position of the eye is in different cases so varied, and so often the consequence of diseased conditions of the brain, the abdominal nervous system, and of the globe itself. Here is a wide range for the production of the disease independently of the cases which are produced by diseases of the orbit. No rational physician will expect to cure such a strabismus by myotomy; and the operation can be applicable only when the malposition of the eye is primarily dependent on the muscle, i. e., when there is a shortening of one of the muscles of the eye, which holds the eye to one side, and limits its motions towards the other; but even here more than one condition must be laid down.

But unfortunately the necessary basis of the operation, pathological investigation, is at present absolutely and entirely deficient; no one has yet clearly convinced himself on the dead body that a shortened state of the muscle exists in squinting. The operation, however, may itself be made the means of investigating the nature of the disease; and here again, as has been the case with club-foot and wry-neck, surgery may be made the assistant of pathology.

With regard to the true orthopædics of the eye, i. e., its acquiring the right position after the operation, this consists in the constant endeavour and active exertion of the patient, to give his eye the proper direction. A club-foot or a wry-neck would just as soon return to its right position after division of the tendons without assistance as a squinting eye will. A good method of insuring success is to tie up the sound eye; the eye that has been operated on then always moves to the centre of the orbit, and the longer it is kept there the more likely it is to remain. In few cases will this or some similar measure be unnecessary.

The author also suggests the propriety of endeavouring to make a subcutaneous or rather a subconjunctival division of the muscle, which he believes would avoid all the inconveniences to which the present method is subject. He has performed it on the dead subject and on animals; and has no doubt it may be applied to actual cases of strabismus.—*Ibid., from Monatsschrift für Medicin,* &c. June, 1840.

———

*On certain Abscesses occurring in different parts of the Body, during the course of Diseases of the Urinary Organs.* By M. Civiale.—M. Civiale has observed that, sometimes during the progress of gonorrhœa and other diseases of the urethra, also in affections of the prostate gland, and when there is stranguary or dysuria, or foreign bodies in the bladder, certain parts of the body, particularly the extremities and the larger joints, are attacked with pain, which may be dull and diffused, or acute and circumscribed, which is at first generally supposed to be rheumatic, but which terminates in the formation of large abscesses, differing in some respect from common phlegmons. M. Civiale states that these cases have escaped the observation of authors, but says that since his attention has been directed to them he has met with numerous examples. When these accidental pains first make their appearance, the patient complains of a peculiar "*empâtement*" (?) and numbness of the part, which come on before any swelling is apparent. These local symptoms are accompanied with lassitude, weakness, anxiety, and general constitutional disturbance which does not correspond at all with the degree of pain, which in the early stages is generally slight and diffused. As the case advances the pain becomes more acute, there is complete loss of appetite and sleep, great prostration of strength, the pulse being sometimes scarcely to be felt; rapid emaciation, dry tongue, troublesome cough, and lastly delirium. The disease often commences with a febrile paroxysm, which may return at regular intervals or be continued. Together wlth these symptoms the urine is often of a deep orange colour, and is excessively fœtid, and when this state of the renal secretion is met with, the case is always very serious. Local redness and swelling of the painful part, with evidence of the formation of matter, frequently do not make their appearance till a late stage of the disease.

One peculiarity in this affection is a tendency in the local disease to shift its position, and it often appears in different places at the same time. The suppuration in distant organs is often accompanied also with the formation of an abscess in the scrotum, perineum, or neighbourhood of the urethra or bladder, which, however, form no communication with the urinary passages.

With regard to the causes of this disease, M. Civiale has not remarked any predisposing circumstances. All the cases occurred in adults. In four there was stricture of the urethra, during the treatment of which affection, (in one case, by cauterization, and in the three others by dilatation,) the abscesses were developed. In a fifth case there was serious disease about the neck of the bladder, and in several others there was urinary calculus, which had been broken down by the operation of lithotrity. In all the cases there seemed to have been some considerable irritation or inflammation of the urethra, or neck of the bladder, which appeared to give rise to the distant affection.

The peculiar state of the system, accompanied by the formation of these abscesses, is attended with considerable danger, and M. Civiale lost the first two patients which he treated.

The treatment found most efficacious was scarification of the whole of the affected surface in the early stage (from which a reddish or yellow coloured serum is generally discharged for several days in considerable abundance,) and then the use of emollient poultices. This plan will sometimes cut short the disease, but if the inflammation has reached the deep-seated cellular tissue of the limb between the muscles, this texture becomes indurated, and a vast abscess soon forms; several large deep incisions must now be made completely through the affected parts which will allow of the escape of the matter, and also unload the distended vessels. The surgeon must not always wait for the sense of fluctuation to be apparent, before he makes his incisions, for great relief will be afforded by cutting through the inflamed and indurated tissues before pus has actually been secreted, and the progress of the disease may be thus arrested. M. Civiale does not make any remarks concerning the general or constitutional treatment of the patient, but we presume that this must be conducted on common principles.—*Ibid., from Bulletin Gén. de Thérapeutique.* Avril, 1840.

---

*New Operation for the Cure of Vaginal Cystocele.* By M. JOBERT, Surgeon to the Hospital of St. Louis.—The following is the substance of a report made to the Royal Academy of Medicine, by a committee consisting of MM. Blandin, Danyan, and Gimelle.

M. Jobert divides his memoir into three parts: in the first, he treats of the history of the disease, and the modes of treatment which have been hitherto employed for its removal. In the second, he proceeds to point out his method of obtaining a radical cure. In the third, he gives some new ideas, based on his pathological researches, with regard to the mode of formation of the cystocele.

Vaginal cystocele was treated of in the work of Leblanc, printed in 1775. Since that time, many surgical authors have noticed it; all have attributed it to a rupture or abrasion of the anterior wall of the vagina, allowing the bladder to pass into the cavity of the former, filling it by its distension, and causing a projection more or less considerable through the os externum. Until lately, palliative means alone have been adopted, which were totally incapable of effecting a radical cure. Pessaries under all forms can be but palliatives.

The well-known operations of Marshall Hall and Dieffenbach have been performed with success by Berard and Velpeau in cases of prolapsus uteri, in which the bladder and vagina have been also drawn down; by these means the displaced parts may be retained within the cavity of the vagina; but in cystocele, M. Jobert says the bladder has an abnormal magnitude; and although it does not project from the vulva, it is nevertheless displaced, resting in such a manner on the perineal surface of the vagina, that the anterior wall of the bladder becomes superior. Thus after the operation just named, though there is not external projection, the organ is not able to regain its ordinary size or position.

M. Jobert considers that in order to effect a radical cure, three things must be done: first, the bladder must be restored to its natural situation; second, the anterior wall of the vagina distended by the tumour must be reduced to its ordinary proportions, so as to maintain the bladder when replaced; but this latter result cannot be obtained by causing a loss of substance in this wall, and reuniting the edges according to the method of Hall, without injuring the orifice of the vulva. The proceeding has also entailed great difficulties in its execution, and possibly severe accidents, therefore the author substitutes, thirdly, the following method: He incloses within two curved transverse lines an oval space, more or less considerable, on the posterior surface of the tumour or the anterior surface of the vagina by means of caustic, so as to form an isolated spot, repeating the application of the caustic until the mucous membrane is destroyed. He then pares the edges with scissors or a bistoury, draws them together, and maintains them in apposition by means of straight needles, the points of which are removed, and a twisted suture.

Jobert operated in this manner on the 23d of July, 1838, on a female aged forty-five, of strong constitution, who presented at the aperture of the vulva a very large cystocele, which elevated the nymphæ and descended above the urethra and clitoris. The tumour was reddish. The patient easily returned and reproduced it. She often experienced acute pains in the abdomen, and the tumour became excoriated from friction. She had frequent desire to pass urine, the emission of which was often difficult. The tumour was five inches in length, from the orifice of the urethra to the neck of the uterus, and eight inches in circumference.

Seven needles were applied; four large, and three small ones. They were lance-shaped, and were contained in sheaths, which alone were left to retain the threads when the suture was made. One of the large needles was applied to each extremity of the oval, formed by the circumscribed space, two others in the middle, the small ones filling up the intervals. A sound of gum-elastic was introduced into the bladder, and left there in order to prevent the accumulation of urine, and the distension of the viscus. The reunion of the edges of the wound arrested the flow of blood, and no accident occurred. Four of the sheaths of the needles which retained the sutures, were removed on the 3d of August; the others fell of themselves on the 5th and 7th. The sound was discontinued on the 20th of the same month. The woman returned to her occupation as a laundress; and at the end of the month of January, 1839, the time at which she was examined, nothing unfortunate had happened.

Since this time, two other cases presenting the same symptoms as that just related, were operated upon in the same manner by M. Jobert, and the result in all the cases has been equally favourable. Two of the committee appointed by the academy were able to testify to this in one case.

But after the cure of these patients, the anterior wall of the vagina underwent a contraction throughout its whole length, and the uterus was proportionally prolapsed. M. Jobert now prevents this inconvenience by applying the caustic in a vertical, instead of transverse direction. In this way the vagina is retained, and remains of its full length, the uterus suffering no displacement.

Jobert has shown by his pathological researches that the anterior wall of the vagina is neither ruptured nor abraded in the displacement of the bladder called cystocele; but that this disease is the result of the relaxation of the superior pelvic fascia, which having been distended by the elevation of the womb in successive pregnancies, loses its elasticity and no longer sustains the anterior wall of the vagina after delivery in a proper manner; and in this case, a fall, an effort, the habit of retaining the urine in the bladder for too long a period and in too great a quantity, are causes sufficient to produce the disease.

Jobert performed the same operation with success in a case of eversion of the mucous membrane of the posterior wall of the vagina; and the committee are of opinion that it may be practised in similar states of the same tissue in other natural passages.—*Ibid.*, *from Bulletin de l'Académie Royale de Médecine.* Mai 15, 1840.

---

*Luxation of the Second Cervical Vertebra, reduced after seven months by a peculiar method.* By M. Jules Guerin.—The author, in a letter to the president of the Royal Academy of Sciences, states that this case resulted from a fall on the chin; but the displacement did not occur till the next day, and he attributed it then to muscular contraction. On this view of the mechanism effecting the luxation, he has employed an analogous mode of reduction, but in the opposite direction; the displacement of the vertebra being consecutive to the rupture of the ligaments, and of part of the articular surface, under the influence of certain muscles, he endeavoured to bring the antagonist muscles into play, and thus gradually produce replacement. This was effected without accident, and the appearances of the luxation successively disappeared; and the young woman, after three months' consecutive treatment, has the neck perfectly straight, and can execute any movement of the head with the greatest facility. This is interesting, as there is much difference of opinion among surgeons as to the propriety of attempting reduction in such cases. In this instance MM. Marjolin and Bouvier thought there would be great danger in the attempt.—*Ibid.*, *from L'Expérience.* Juin 18, 1840.

---

*New Operation for Prolapsus Ani.* By M. Robert.—Relaxation of the sphincter ani being the cause of this disease, all the remedies hitherto employed for its cure are inefficacious when it arrives at the last stage, as they can only act on the mucous membrane of the rectum. These reflections have induced M. Robert to shorten the sphincter in proportion to the amount of relaxation, so that the two cut surfaces of the muscle might unite and form a narrow ring to oppose to the descent of the mucous membrane. This operation was performed with success on a washerwoman, thirty-three years of age, in June, 1839, In the hospital of La Pitié. This woman, in her third pregnancy, had a prolapsus ani, which was only temporary, though it caused some pain. Her fourth pregnancy produced a prolapsus uteri, a permanent and considerable prolapsus ani, with relaxation of the abdominal walls. M. Robert excised a portion of the mucous membrane with some temporary relief; but the disease afterwards increased, the discharge of fæces became involuntary, and she suffered from pains in the loins and upper part of the thighs. When she entered the hospital the sphincter was so much relaxed that four fingers could be easily introduced.

The patient having been prepared for the operation by progressive diminution in diet, and the use of opium, in order to effect long-continued constipation, M. Robert proceeded to operate in the following manner: An incision was made on each side of the anus, each incision being commenced a few lines external to the orifice, and carried backwards towards the coccyx. The fold of the integument between the incisions, together with the portion of sphincter it covered, were removed, and the muscle was thus shortened by half its length. The wound was united from one side to the other by three points of suture. On the sixth day after the operation the sutures were removed. Union was nearly complete, but a fistulous passage remained from the anus to the coccyx. On the fifteenth day the woman had not passed any fæces. On the next day, the want of defecation being felt, in order to prevent any straining, the bowels were relieved by the curette. On the forty-first day the patient, who before the operation could not retain her fæces, kept an injection during the whole day; there was no more prolapsus; the opening had become of the ordinary size; but the finger, when introduced, did not experience the energetic contraction of the sphincter which occurs in the normal state. With the exception of a slight protrusion of mucous membrane, the cure was complete in August.—*Ibid., from Gazette Médicale.* Juin 20, 1840.

*Dislocation of both radio-carpal articulations. The left carpus backward, the ulna being also dislocated from the right radius: the right carpus forwards, ulna not dislocated from radius, unattended with fracture.* By N. J. HAYDON, M.R. C.S.—John Davey, aged thirteen years, applied to Mr. R. Rudall, of Sheepwash, on June 11th, 1840. He had been thrown very violently from a horse, and fell on the upper part of the palms of both hands, and on his forehead. On examining him we found a lacerated wound of the scalp, about two inches in length, inclining from the mesial line to the left eyebrow: the left wrist presented a considerable protuberance on its anterior aspect; the styloid process of the radius no longer had its position opposite to the trapezium, but was thrown before the carpus, and took up its residence on the scaphoid and trapezium: the ulna was dislocated from the radius, and rested on the unciform bone; the forearm was slightly bent on the humerus; the fingers similarly flexed on the hand throughout their articulations. During the reduction of this wrist the patient complained of no pain.

The right wrist presented a very considerable tumour on its posterior aspect, occasioned by the presence of the carpal head of the radius and ulna, and a very irregular knotty tumour terminating abruptly on its anterior aspect, caused by the presence of the bones of the carpus. The forearm was very considerably flexed on the humerus, and in a state between pronation and supination; the thumb strongly abducted; the metacarpal phalangeal articulations in a state of the greatest extension on the metacarpus; the two extreme joints slightly flexed. There was a strong aversion to have the arm moved, the slightest motion causing extreme agony: there existed in this arm no dislocation of the ulna from the radius. A very careful examination was made to determine what parts came in contact with the resisting force. We found very extensive bruises on the palms of both hands, but not the slightest on the back of either hand. We could not obtain the slightest evidence of any fracture existing; and recollecting the opinion of Dupuytren,* "that there was not a single unequivocal instance on record of a dislocation of the radio-carpal articulation;" and, "that he invariably found these pretended accidents always turned out to be fractures of the radius near the articulation," we were very careful in our examinations. Moreover, we were strengthened in our opinion that these were cases of dislocation unattended with any fracture, because the dislocations appeared so perfect, the two tumours in each member so distinct, the reduction so complete, the strength of the parts so great after reduction, the very trifling pain after reduction. Within an hour after the dislocation was reduced the patient could rotate the hand, and supine it when prone. This could not, we believe, have been done had there existed a fracture; and lastly, in the right arm the radius retained its exact parallel relation to the ulna, extending a little beyond it. On the left the ulna was carried a little inwards and forwards, and so remained after the radio-carpal dislocation was reduced. We should not have reported this case had it not seemed important, and one of very rare occurrence; for we find in one person from the same accident, *and from a force applied in the same manner*, one presenting a dislocation of the carpus backward, the other presenting the carpus dislocated forward.

Sir Astley Cooper, whose long experience gives to all his opinions great weight and value, states at page 502 of his work on Dislocations and Fractures, that "the dislocation of both bones is not of very frequent occurrence; but when it does happen the bones are thrown either backwards or forwards, according to the direction in which the force is applied." "In the dislocation of the radius and ulna *backwards*, the person falls upon the *back* of the hand, the radius and ulna are thrown upon the posterior part of the carpus, and the carpus itself is forced under the flexor tendons."

---

* In addition to the two cases given by Desault and Boyer, there is one lately published by M. Mallé in his Surgical Clinique, in which a post-mortem examination demonstrated perfectly the possibility of this injury. A specimen in St. Bartholomew's Hospital exhibits the same fact. The case of M. Mallé we extract from a review of his work in the British and Foreign Medical Review:

"A soldier, while tipsy, threw himself from a second floor into the street. He fell, to all appearance, partly on the palm of his right hand, and partly on his head. When carried to the hospital he had all the symptoms of severe concussion. The hand appeared at first sight considerably deformed; it was extended on the forearm, and the carpo-metacarpal region appeared shortened; at the anterior part of the joint there was a remarkable projection, a little convex transversely, and apparently formed by the carpus. The radius and ulna were placed behind; the former preserved its length, and as usual extended beyond the ulna. The fingers were flexed, the tendons of the flexor muscles appearing very tense. With considerable difficulty the dislocation was reduced, but the patient died of the effects of the concussion three days after the fall.

"All the parts around the wrist were found engorged and infiltrated with blood. The anterior ligament of the capsule was ruptured; the lower extremity of the radius was unbroken, but the bones of the first row of the carpus were unnaturally moveable upon one another; the ligaments uniting them seemed to have suffered from some violence, but there was no trace of fracture. At the posterior or dorsal part of the wrist there was a similar bloody infiltration of the soft parts, but no injury of the bones."

Dupuytren made some curious calculations as to what force would be necessary to effect a dislocation of the radio-carpal articulation, and states his most entire disbelief in the possibility of the accident. Baron Boyer, p. 256, vol. iv. of his "Maladies Chirurgicales," states, "notwithstanding the violence of the efforts, capable of overcoming the resistance of the tendons and ligaments, so as to produce a luxation of the hand, this luxation can take place in four different ways; to wit, backwards, forwards, inwards, outwards. The luxations backwards and forwards are by far more frequent than those inwards or outwards."

The rarity of an accident of this nature induced me to forward a copy of this case to Sir A. Cooper, who suggests—"Is it not possible this patient might have fallen on the back of one hand and the palm of the other?" To this I can only answer, that the extensive bruises and laceration on the palms of either hand, more especially on the ball of the right thumb, while there is not the slightest bruise or laceration on the back of either hand, seem to afford fair evidence where the resisting force was applied. Respecting the opinion of Dupuytren, Sir A. Cooper says, "that Dupuytren was undoubtedly wrong, although the dislocation is rare."

The treatment of our patient was very simple. The left wrist was speedily reduced. The elbow being fixed, extension was made from the hand, at the same time gradually flexing the hand on the forearm. The right wrist, the elbow being fixed, was reduced by firmly grasping the thumb, the operator's thumb being applied over the outer part of its metacarpal bone, while his index-finger firmly embraced its ball; gradual and direct extension was maintained for several minutes; when the carpus resumed its position, a very distinct and audible "*click*" was noticed by several persons present. Splints, including the forearm and hand, with roller bandages, were applied, and retained for eighteen days, the parts being kept cool with evaporating lotions. The progress of the patient was altogether favourable.

*June 24th.*—Attentively examined both wrists this morning: the patient remains free from pain, there is no deformity: considerable swelling and stiffness still continue, the usual effects of extensive laceration and spraining of the ligaments. Splints and bandage again applied, and retained until June 28th, when they were discontinued, as the swelling had nearly subsided, and the wrists had regained sufficiently their power to admit of all the usual movements of the hand being freely exercised; a bandage was, however, still retained on the right wrist.

*July 16th.*—We saw the boy this day at his usual work on the farm; complained of no pain, but considerable weakness. There remain a stiffness and some enlargement around the joints, but this appears to be gradually subsiding. The bandage was left off to-day, and the patient told to have his wrists pumped upon several times daily.—*London Medical Gazette.*

---

*Coincident Occurrence of Variola, Modified Variola, and Varicella in the same Individual.* By Dr. Brunzlow, of Brandenburg.—One of the author's family, a child a year and a half old with a very irritable skin, who had been vaccinated when a few months old with lymph taken directly from the arm of a healthy child, and who had had five regular vesicles on the two arms, was affected during its infancy with various exanthemata. It had scarcely recovered from an attack of urticaria when the diseases mentioned above began to appear, though the child had not been in contact with any one affected by them. The small-pox eruption occurred in a measure regularly; it commenced on the face and finished on the extremities, so that within a few days the whole body was covered with an innumerable quantity of pustules.

When the eruption was completed, and when, according to the general rule, no new pocks ought to have broken out, there appeared both on the face and on the trunk and extremities, and between the vesicles that were already formed and were passing through their regular course, a number of others far smaller, paler, and possessing less activity, which both in their form and in their course were very different from those that first appeared, and which from all their characters could be regarded only as varicella.

The vesicles first mentioned went on in their development, increased in size, became filled with a serous fluid, presented depressions at their centres, and became surrounded with a red areola; but the author observed that after the fourth day of their eruption, the majority of them did not go on to suppuration. Their redness diminished, the lymph which they contained dried up, and after a few days they were covered with crusts. They presented evident examples of modified small-pox, the varioloid disease.

Several of the vesicles of the first eruption, however, did not dry up, but went on further in their development. They filled with pus, their areolæ reddened and were vividly inflamed, and after a few days they acquired the form of genuine and perfect small-pox. Their number, however, was not considerable; there were few on the face, but they were more numerous on the abdomen and on the inner side of the forearm and thigh.

The fever accompanying the eruption, which did not diminish on the drying of the varioloid eruption, increased with the progressive suppuration of these last pustules, and became rather severe.

The varicella, while the variolous eruption was suppurating and the varioloid was drying, had not yet finished coming out; and new vesicles of it kept constantly appearing among those of the other diseases, and after lasting a few days and filling with a watery lymph, dried and became covered with a fine light scurf.

At last when the variolous eruption had completed its stage of suppuration and passed into that of drying, and when the desquamation of the varioloid was finished, the fever diminished, no more vesicles of varicella appeared, the patient became more lively, and regained his appetite, and soon after was completely restored to health. The diseases had altogether occupied some weeks. The scars which remained from the variolous eruption were quite different from those of the varioloid; those on the face still indicate their origin by their characteristic signs. The scars of the varicella, on the contrary, disappeared very soon after the disease, and those of the varioloid eruption have been lost sight of for many years, so that now no trace of either of them can be discerned.

[The author has added some rather lengthened observations; but they are not sufficiently apposite to his singular though somewhat loosely related case to render their translation necessary.]—*Brit. and For. Med. Rev.*, *from Medicinische Zeitung*. Mai 20, 1840.

*Case of Menstruatio Recidiva.* By Dr. Petersen.—A healthy woman, aged seventy-nine, was seized on the 26th of March with uterine pains; these lasting a few days were terminated by a hæmorrhagic discharge. On the 23d of April she was again affected in the same way, the discharge appearing on the 25th, and continuing four days. Since that period (twelve months ago) she has regularly *menstruated.*

[There are several cases on record in which the menstrual periods have been regularly continued until seventy, eighty, and ninety years of age. It has been said (Locock) that such cases are not genuine cases of menstruation, but sanguineous discharges arising from uterine disease. But we have at this moment a lady aged sixty-two under our care, who regularly menstruates without any symptom of uterine disease. A most extraordinary case, similar to that of Dr. Petersen's, is given by Velasquez, of Tarentum, of the abbess of Monvicaro, who at the age of one hundred, after a severe illness, had a recurrence of the catamenia, and not only this, a new set of teeth and a fresh head of hair appeared!—*Ibid.*, *from Bibliothek for Læger.* Feb., 1840.

*Anatomico-Pathological Researches on Cirrhosis of the Liver.* By Alfred Becquerel, Paris.—The name cirrhosis was given by Laennec to a peculiar affection of the liver in which that organ appears as if filled with numerous yellow granules, which are sometimes present in such numbers that the surface of a section of the liver presents an uniform yellow colour. If this surface is carefully examined, M. Becquerel says an innumerable quantity of minute granulations may be discovered, which may be compared to the lobules of hardened and reddish-coloured fat which are commonly found in the subcutaneous cellular tissue of the thigh and leg of anasarcous subjects. These little masses are sometimes intimately united to the texture of the liver, but they are frequently separated from it by a thin layer of cellular tissue which forms a slight envelope round the globules from which they are easily detached.

The exact nature, the causes, and the symptoms of this affection are very imperfectly understood; and it is for the purpose of elucidating these points that M. Becquerel has published the present memoir. Laennec considered that in this disease a new morbid product was deposited in the liver, which he termed "cirrhose," and which he believed might be also developed in other organs of the body; appearing first in a state of crudity, and afterwards softening. Andral states that the granules, in his opinion, merely arise from hypertrophy of the white or secreting part of the liver, which in the more advanced stages is accompanied with corresponding atrophy of the red or vascular tissue, giving rise to that shrivelled state of the liver generally which was described by Laennec as frequently occurring in this disease. M. Cruveilhier considers that cirrhosis consists in atrophy of a great number of the lobules of the liver, and a corresponding degree of hypertrophy of those which remain which causes the irregular granulated surface. Dr. Carswell, who gives an admirable description and delineation of this disease in the article Atrophy, in his work on Pathological Anatomy, regards it as consisting in atrophy of the lobular structure of the liver, produced by the pressure of a contractile fibrous tissue formed in the capsule of Glisson; an opinion which, as we shall find, very nearly coincides with that adopted by the author of the present paper. M. Bouillaud regards cirrhosis as a disunion of the two natural elements of the liver, the yellow granulations being nothing more than the secreting lobules undergoing disorganization in consequence of obliteration of the vascular tissue, and consequent obstruction of the hepatic circulation.

M. Becquerel, previously to stating his own opinion on the nature of the morbid alteration of the liver in cirrhosis, briefly reviews the different opinions concerning the intimate anatomical structure of this organ in its natural state; and he adheres to the notion that there are two distinct substances: one yellow, which is the true secreting tissue, and the other red, which consists of the ramifications of veins and arteries. He inclines to the opinion of Cruveilhier, that each lobule possesses a spongy tissue not injectible; and thus widely differs from Mr. Kiernan, who describes the liver as being throughout essentially vascular; the lobules consisting of minute radicles of the biliary ducts surrounded by a plexus of the terminating branches of the portal veins. The latter eminent pathologist explains the differences in appearances between the red and yellow parts of the liver by the different degrees of injection of the different portions of its tissue.

Admitting, then, the presence in the liver of secreting lobules, into the interior of which no vessels enter, not even the minute ramifications of the biliary ducts, which together with the branches of the vena porta and hepatic artery form a network round the lobules without penetrating them; the lobules themselves merely consisting of a mass of albuminous corpuscules, as described by MM. Dujardin and Verger; M. Becquerel next endeavours to ascertain which of the tissues—the secreting or the vascular—is the one altered in cirrhosis, and he arrives at the conclusion that it is the latter only. He says that this tissue becomes infiltrated with a yellowish plastic or fibro-albuminous matter, which possesses the properties which characterize fibrine and albumen; for it is coagulated by heat, and by a continued application of that agent is converted into a horny substance. He considers that this effused matter is analogous in its composition to the false membranes which are developed in mucous, and more frequently serous, tissues; and he says that it undergoes the same changes.

M. Becquerel has determined that in this disease of the liver no pus globules can ever be detected in the altered structure; a very small quantity of fatty matter is likewise present, which is always abundant where pus is met with. He deduces from these facts that cirrhosis does not arise from acute inflammation: the absence of fat will also distinguish this disease from the fatty disease of the liver, in which this organ is likewise of a yellow colour.

This fibro-albuminous matter is supposed to be deposited by interstitial infiltration in the central part of the secreting lobules, and the yellow substance of the liver becomes thus hypertrophied, and causes compression, and eventually atrophy of a great part of the red or interlobular substance. Many of the vessels and ducts are thus obliterated, and the tissue of the liver becomes difficult of injection. In the most advanced stages of this disease the effused matter loses great part of the water which entered into its composition, becomes firmer, and contracts in size. It thus undergoes the same changes as false membranes, which are at first soft and thick like white of an egg, but which afterwards becomes organized and firm, and lessen in volume. From the contraction of this matter the yellow substance of the liver which was at first hypertrophied becomes atrophied, the altered lobules are diminished in volume, and many of them often coalesce, forming the patches which were described by Laennec as occurring in certain forms of this disease, and which give an irregular appearance to the surface or to a section of the liver. When this retraction has taken place, a great portion of the red or interlobular tissue of the liver no longer exists, and we only see an irregular agglomeration of yellow tubercles of different sizes, and the whole organ becomes hardened and shrivelled.

The immediate causes of this disease are completely unknown. M. Becquerel conjectures that it may arise from active and long-continued congestion of the liver, consequent upon chronic disease of the lungs or heart, (with the affections of which organs cirrhosis is often complicated,) or arising from other causes.

Cirrhosis never affects the liver partially; it always attacks all parts of its substance simultaneously. The gall-bladder is almost always quite natural. M. Becquerel has not made any comparative analysis of the state of the bile in this disease, but he found it vary considerably in consistence and colour.

This affection of the liver is mostly complicated with organic disease of other parts, as Bright's disease of the kidney, and various lesions of the heart and lungs; it is rarely met with by itself: M. Becquerel only found seven out of forty-two cases which he could consider as uncomplicated. He classes these complicating diseases under two heads: *First*, Chronic diseases, developed before or at the same time as the cirrhosis. *Second*, Diseases more or less acute, and probably consequences of the cirrhosis. 1. To the first class belong the following:—Lesions of the *Heart*. These are more commonly met with in connexion with cirrhosis than any other; for in forty-two subjects examined the heart was affected with chronic disease in twenty-one cases or in half; and the disease in all these instances had advanced to such a degree that it must have preceded, if it did not give rise to, the disease of the liver. The disease of the heart in these cases is often complicated with other affections, as emphysema of the lungs and chronic bronchitis, tubercular disease, or Bright's kidney.

It is exceedingly difficult to determine whether the affection of the heart gives rise to that of the liver, and if so in what manner. M. Becquerel has no doubt that the former precedes the latter in almost all cases, and supposes that it may give rise to it by keeping up a state of continued congestion of the liver. Pulmonary emphysema and chronic bronchitis, which are sometimes found accompanying cirrhosis, without disease of the heart, may be supposed to act in its production in the same manner, by obstructing the venous circulation. Cirrhosis is very rarely found in connexion with tubercular disease of the lungs. The fatty degeneration of the liver, on the contrary, is a frequent complication of consumption.

The morbid alteration of the *kidney*, known by the name of Bright's disease, frequently accompanies cirrhosis, having been met with fifteen times in forty-two cases. Some differences were observed between these cases of considerable interest: thus in nine cases where cirrhosis and renal disease existed together and appeared to be of about the same standing, old organic lesions of the heart and lungs were also found, which our author considers to have been the exciting causes of the diseases of both the liver and kidneys, repeated congestion of both these organs having been occasioned by obstruction to the circulation. In another series of cases, which includes three out of fifteen, the cirrhosis seemed to be of a much older date than the affection of the kidneys, and probably gave rise to it by obstructing the circulation of the abdominal veins. In the remaining three cases the disease of the liver and kidneys seemed to be of about the same date, and had been probably developed from a similar cause, the nature of which, however, was unknown.

2. In the second class may be arranged the following:—*Ascites*, which constantly accompanies cirrhosis in the more advanced stages; *Peritonitis*, producing purulent effusion and the formation of false membranes; *Pleuritis* and *hydrothorax; Pericarditis*—this has only been met with in a few cases; *Anasarca*—this generally occurs in complicated cases along with ascites, and when the disease of the liver is in an advanced stage. Besides these, *œdema of the lungs*, *pneumonia*, and *pulmonary apoplexy*, have been met with, as well as congestion of the mucous membrane of the alimentary canal; and *intestinal hæmorrhage*, which must be considered as an effect of the disease of the liver, arising from the obstruction of the circulation of the blood through the vena porta.

Cirrhosis is more frequently met with in men than in women: thus of eighteen cases of the simple or uncomplicated form of this disease, twelve occurred in males and six in females. Of the whole number of forty-five cases examined by M. Becquerel, twenty-eight were found in men and seventeen in women.

With regard to the influence exerted by age, the eighteen simple cases may be thus arranged:—

| | | | | | |
|---|---|---|---|---|---|
| Between 18 | and | 20 | years. | | 1 |
| " | 20 | " | 30 | " | 2 |
| " | 30 | " | 40 | " | 7 |
| " | 40 | " | 50 | " | 3 |
| " | 50 | " | 60 | " | 5 |

Thus persons between thirty and forty seem to be most liable to be attacked by cirrhosis. This disease is also met with in children; and M. Becquerel is disposed to think that it occurs more frequently in young subjects than has generally been supposed.

With regard to the symptoms produced by cirrhosis, none exists sufficiently characteristic to enable the physician to diagnosticate its presence during life. The most constant symptom of this disease in its more advanced stages is dropsy, which always commences in simple cases in the abdomen; its approach is generally unaccompanied by pain, but in a few cases the belly is the seat of pain, either with or without pressure, and the presence of peritonitis is thus indicated. Anasarca sometimes precedes the ascites, but Bright's disease of the kidney or disease of the heart are then always indicated. M. Becquerel says that the state of the urine is always peculiar in this disease; it is of a deep orange and often reddish-yellow colour, very dense, strongly acid, and deposits an unnaturally large precipitate of urate of ammonia, which is of a bright red or cinnabar colour. The causes of this state of the urine are unknown.

The course of this affection is uniform and continued, and it always proceeds more or less quickly to a fatal termination, being apparently uninfluenced by any plan of treatment.—*Ibid.*, *from Archives générales de Médicine.* Avril et Mai, 1840.

---

*Complete absence of Menstruation.* By M. Kruger-Hansen.—A woman of about fifty-eight years of age, of a robust constitution, and who had always enjoyed good health, much younger in appearance than she really was, and in whom all the feminine characters were well developed, never in the whole course of her life had any discharge at all similar to the menses. She never had *fluor albus*, or any abnormal sanguineous discharges or sweats, to compensate for the want of the usual monthly secretion. It is added that the sexual appetite was present, but that she never had borne children. M. Kruger-Hansen, from this single imperfect case, draws the conclusion, that the menstrual discharge is not absolutely necessary to woman. It is obvious, however, that, before such a conclusion could be drawn, it would be necessary to ascertain whether all the organs were present, and well developed. The omission of this renders the case incomplete.—*Edinburgh Medical and Surgical Journal, from Graefe's und Walter's Journal*, Vol. xxvi. *No.* 3.

---

*Three cases of Acute Inflammation of the Aorta.* By Dr. Thierfelder.—1. A mason, thirty-five years of age, complained for some days of palpitations, oppression, and sensation of burning heat in the breast, and a dry troublesome cough; and awoke on the 5th of November with great increase of all the uneasy symptoms. On the arrival of Dr. Thierfelder, half an hour afterwards, he was found insensible, with stertorous very slow breathing, and cold extremities. In a short while he came to himself, and muttered a few unintelligible words. His body was swollen, of a bluish hue, and covered with a cold sweat; his pulse was very weak and soft; the action of the heart scarcely perceptible; and the respiration so slow that he scarcely breathed five times in two minutes. Fits of suffocation returned at intervals, and he died about three hours after being seen.

The lungs were found adhering to the pleura at some points, but were otherwise healthy, and no fluid was met with in the cavity of the pleura. The pericardium contained nearly fifteen ounces of serous fluid. The heart was filled with blood, the right auricle enlarged, but the ventricles appeared healthy. The *vena cava* and pulmonary veins were much distended with venous blood, but no morbid alterations were observed on them.

The ascending aorta presented the marks of violent inflammatory action on its inner coat; it was highly reddened and injected, and this appearance extended to its numerous ramifications; while numerous patches of plastic lymph adhered strongly to its surface.

The other organs were healthy.

2. An unmarried woman, twenty-three years of age, was seized on the 20th August, 1830, with a violent rigour, caused by a fright; followed by anxiety, oppression, and sensation of burning heat in the chest, violent cough, and bloody coloured expectoration, and considerable fever. Two days after this, palpitations of the heart, and fits of suffocation, with a constant dry cough, were complained of. It was then she was seen by Dr. Thierfelder. She had great anxiety, and was constantly groaning, and complained of a burning sensation and feeling of oppression at the upper part of the sternum, stretching towards the left clavicle, the neck, and the back. The respiration was hurried, and from time to time violent attacks of coughing came on, with fits of suffocation. The face was red, and somewhat swollen, and the pulse was one hundred and sixty in the minute, hard, but very small. She died on the night of the 22d.

The lungs were found healthy, only containing in their bronchial tubes a quantity of reddish frothy mucus. The pericardium contained nearly a pound of serous fluid. The heart was distended with black blood, but especially the right side, which was also paler and softer than usual. The left side, both auricle and ventricle, was firm, and appeared somewhat thickened. The internal membrane of the left ventricle of the heart, as well as that of the aorta, from its origin till opposite the third dorsal vertebra, as well as that of the coronary arteries, was reddened in consequence of inflammatory action. The internal membrane was considerably swollen in the aorta, so much so, that about a third of the diameter of the vessels was lost, and its surface was covered with an effusion of plastic adherent lymph.

The other organs of the body appeared to be healthy.

3. A clerk, twenty-eight years of age, on the 24th of January, after washing himself with cold water, and sitting writing for several hours in a cold apartment, became chilled. He passed a restless night, and complained of rheumatic pains in his left arm and head. Next morning fits of suffocation came on, with a sensation of oppression and heat, together with a painful pulsating feeling at the upper part of the sternum, and of the left clavicle, stretching through to the back. The pulse was very quick, hard, and tense; the respiration was rapid; and he had troublesome fits of coughing. The abdomen was swelled, and painful to the touch: his tongue was dry, and he had considerable thirst.

Towards evening he got worse; the fits of suffocation were more severe; his body was swollen; his eyes injected and nearly insensible to the approach of light; his respiration very laborious; and his thirst almost unquenchable. He had also delirium. In addition to these symptoms, on the evening of the 26th he had ringing in his ears, which was much increased during the fits of coughing.

On the morning of the 27th these symptoms began to abate. The oppressive sensation of heat and palpitation still remained: the pulse was still one hundred and seventy in the minute, hard and tense. Towards midnight, however, a copious warm perspiration, as well as a red miliary eruption, broke out over the body, and from that period he gradually recovered.

Acute inflammation of the aorta is always a disease difficult to distinguish. Dr. Thierfelder considers the peculiar cough which attends it sufficient in most cases to declare the nature of the malady. He always found it very frequent and very hard.—*Ibid.*, *from Ammon's Monatschrift*, April, 1840.

---

*On the Etiology and Treatment of Congenital Luxations and Pseudo-Luxations of the Femur.* By Dr. J. Guerin.—I have established it as a fact, in my History of Deformities of the Osseous System, that the greater number of congenital articular contractions are the product of primitive muscular retraction. . . . . . . . . . I conclude: 1. That congenital luxations of the hip-joint are, like club-foot, torticolis, and spinal deviations, the product of primitive muscular retraction, and that the kind of luxation depends upon the direction in which the retraction happens. 2. There exists an order of deformities of the hip-joint not hitherto indicated, and to which I apply the term *pseudo-luxations*, because they look like luxations, although the head of the bone remains in the cotyloid cavity. These vary as do the complete luxations, and from the same causes. 3. The essential treatment consists in division of the retracted muscles. I have thrice performed this operation with success. The congenital luxations and pseudo-luxations of joints depend in the majority of cases on primitive muscular contraction, which takes place in three different modes: by shortening, by paralysis, and by stoppage of development of the retracted muscles; and the different varieties of these deformities are, like those of the neck, the spine, and the foot, the effect of this retraction differently distributed in the muscles of these parts. In a girl aged fourteen years, I divided the biceps, semi-tendinous, semi-membranous, and rectus internus, for two incomplete luxations of the knee, produced by primitive retraction of these muscles; on each side there was subluxation of the tibia backwards upon the condyles of the femur, rotation of the limb outwards through the space of about one-fourth of a circle, and an inclination outwards of the leg upon the thigh of about sixty degrees. The rotation outwards, the lateral inclination and the gliding backwards of the tibiæ, were reduced on the day following the operation to the simple normal flexion of the leg upon the thigh, and since that period there remained nothing more than a certain degree of permanent flexion of the joint. In order to convince those who feel any doubt respecting the safety with which these operations may be performed, I may add that, on the same day, I successively divided in the young female above mentioned thirteen muscles or tendons beneath the skin, for the relief of certain deformities from which she suffered; on the following day she felt no kind of pain nor indisposition, nor were there any symptoms of inflammation in any one of the divided parts.—*Ibid.*, *from Gazette Médicale de Paris.* No. iv. 1840.

---

*On Phlebitis Hepatica Neonatorum.* By Dr. Schöller, Assistant Physician at the Obstetric Clinic, Berlin.—The first case related by Dr. Schöller is that of an infant, healthy for the first nineteen days of its life, when, after exposure to cold, it refused the breast, the bowels ceased to act, the abdomen became painful and tympanitic, the child vomited a greenish fluid, and died within twelve hours after it showed the first signs of indisposition.

On a post-mortem examination were found a puriform effusion in the abdominal cavity, and thick layers of lymph coating the stomach, spleen, omentum, and colon. At the point where the vena portæ and vena umbilicalis enter the liver, they were surrounded by thick layers of yellowish fibrine. On opening the vena portæ thick pus exuded; and the walls of the vessel, even in its finest ramifications, were so much thickened as to communicate to the liver considerable firmness when divided. The gall-bladder was pale, and contained a greenish mucous fluid. The spleen was pale and friable. The intestines were full of a green fluid, similar to that which the child had vomited during life.

In the second case the disease ran a still more rapid course. A female child was strong and healthy up to the twentieth day after its birth. It then refused the breast, its respiration became hurried and gasping, the abdomen hard and painful, and the child vomited a greenish fluid. The same evening trismus and convulsions came on and recurred at intervals, till, in the course of the night, the child died. An examination disclosed extensive peritonitis with effusion into the abdominal cavity, and the umbilical and portal veins were thickened and distended with pus.

On the right side was an inguinal hernia, the sac of which extended into the labium, and contained the red and swollen Fallopian tube. The unusual shortness of the round ligament of the right side seemed to have been the cause of the hernia of the tube; for the uterus had been drawn by the round ligament so forcibly to the right side as to give to its long axis an oblique direction.—*Ibid.*, *from Neue Zeitschrift für Geburtskunde.* Band vii.

---

*On the Radical Cure of Inguinal Hernia by the Horizontal Position, after the plan of M. Ravin.* By M. Biagini.—A man, thirty-two years of age, consulted M. Biagini on account of a tumour in the left inguinal region, which this physician considered to be an entero-bubonocele, and which had resisted all efforts at reduction. M. Biagini proposed a long continuance in the horizontal posture to the patient, aided by compression, but he refused it. A year afterwards the man was obliged to put himself under treatment for articular rheumatism complicated by pericarditis. After three months' decubitus, which was consequent on the rheumatic affection and its treatment, the hernia did not exist, nor could it be made to appear by any effort or coughing. M. Biagini felt assured that the left external inguinal ring was singularly contracted. He considered this case as confirmative of the views of M. Ravin, and drew the following conclusions: 1. It is possible to obtain a radical cure of some herniæ by a long continuance of the horizontal position. 2. The cure takes place by a diminution in the size of the canal and the retraction of its walls, by which the lost obliquity is restored. These changes occur solely by the *vis tonica* of the tissues. 3. Though the tissues possess sufficient *vis tonica* to bring about the desirable changes, it is necessary that the general strength of system of the patient be somewhat considerable.—*Ibid.*, *from Bulletino delle Scienze Mediche.* Gennario, 1840.

---

*Colocynth Oil, a substitute for Croton Oil in Neuralgia, Sciatica, &c.* By M. G. Janelli.—Dr. Janelli mentions that he has found the oil of colocynth a valuable and cheap succedaneum for croton oil, as an external application in neuralgic affections, but especially in sciatica. He relates in detail three cases of its efficacy in sciatica, and three in cases of rheumatism. After frictions with the oil, the patients generall fell asleep, so much were their sufferings alleviated, and in a few days were so far recovered as to be enabled to return to their usual avocations.—*Edinburgh Medical and Surgical Journal, from Observatore Medico*, January, 1840.

